# Hepatocyte growth factor produced in lung fibroblasts enhances non-small cell lung cancer cell survival and tumor progression

**DOI:** 10.1186/s12931-017-0604-z

**Published:** 2017-06-15

**Authors:** Nobuhiro Kanaji, Masanao Yokohira, Yuko Nakano-Narusawa, Naoki Watanabe, Katsumi Imaida, Norimitsu Kadowaki, Shuji Bandoh

**Affiliations:** 1 Department of Internal Medicine, Division of Hematology, Rheumatology and Respiratory Medicine, Faculty of Medicine, Kagawa University, 1750-1 Ikenobe, Miki-cho, Kita-gun, Kagawa, 761-0793 Japan; 2 Onco-Pathology, Department of Pathology and Host-Defense, Faculty of Medicine, Kagawa University, Kagawa, Japan

**Keywords:** Lung cancer, Fibroblast, Cancer-associated fibroblast, Hepatocyte growth factor, PHA-665752, Met, Xenograft, Tumorigenicity

## Abstract

**Background:**

The influence of lung fibroblasts on lung cancer progression is not fully understood.

**Methods:**

Lung fibroblasts (HFL1, MRC5, and IMR90 cells) and non-small cell lung cancer (NSCLC)-derived cell lines (A549, EBC1, and HI1017) were cultured under serum-free conditions, and the resulting culture media were designated “cell-conditioned media”. Cell survival (viability) was assessed by WST-1 assay. Concentrations of hepatocyte growth factor (HGF) were measured by ELISA. The BALB/c-*nu* mouse strain was used for the xenograft model.

**Results:**

Lung fibroblast-conditioned media enhanced the survival of the three NSCLC cell lines tested. HGF was produced to a greater extent by lung fibroblasts than NSCLC cells. Exogenous HGF enhanced the survival of NSCLC cells. Either an anti-HGF neutralizing antibody or the Met inhibitor PHA-665752 inhibited the fibroblast-conditioned media-enhanced survival of NSCLC cells. The co-inoculation of mice with NSCLC cells and fibroblasts enhanced tumorigenicity and tumor progression in a mouse xenograft model. PHA-665752 significantly inhibited tumor progression that occurred after the co-inoculation of NSCLC cells and fibroblasts. In addition, HGF production by fibroblasts was stimulated by NSCLC cells.

**Conclusions:**

The current study provides evidence for an interaction between fibroblasts and NSCLC cells via the HGF/Met signaling pathway, which affects NSCLC cell survival and tumor progression. These findings may contribute to the development of anti-cancer-associated fibroblast therapeutic strategies.

**Trial registration:**

No trial registration is required because this study is not a clinical trial. This study does not include any participants or patients.

## Background

Recent evidence has shown that the cancer microenvironment plays a significant role in cancer progression [1]. Although most host cells in the cancer microenvironment possess certain cancer-suppressing abilities, the microenvironment changes during the progression of a malignancy and eventually promotes growth, invasion and metastasis [2]. Fibroblasts represent an abundant cell population in the cancer microenvironment [1]. These fibroblasts, which are termed cancer-associated fibroblasts (CAFs), stimulate cancer cell proliferation, differentiation, invasion, and metastasis. They do this the production and alteration of the extracellular matrices, which function as a scaffold for cancer cells. CAFs also produce different types of growth factors and cytokines, such as fibroblast growth factor, interleukin-6, and hepatocyte growth factor (HGF) [3, 4].

HGF participates in a number of biological activities, and its binding to Met, a heterodimeric receptor tyrosine kinase, induces its phosphorylation and stimulates downstream signaling pathways including the mitogen-activated protein kinase, phosphatidylinositol 3-kinase-Akt, and signal transducer and activator of transcription three pathways [5]. These pathways are known to involve cell proliferation, migration, invasion, angiogenesis, and survival [5, 6]. The overexpression of Met has been reported in many types of cancer including lung cancer [5, 7], and in one study, fourteen of 23 non-small cell lung cancers (NSCLCs) (61%) showed strong expression of Met [8]. It has also been reported that most (82-89%) NSCLC cell lines express Met [8, 9]. Moreover, the significance of the HGF/Met pathway on lung cancer progression has increasingly been reported [9–14].

Based on this perspective, we investigated the interaction between lung cancer cells and lung fibroblasts within the context of the HGF/Met pathway and its impact on lung cancer progression. We found that HGF production within lung fibroblasts was stimulated by lung cancer cells and that HGF promoted the survival of lung cancer cells. The inhibition of Met suppressed tumor progression in tumor xenografts that developed after the co-inoculation of mice with lung cancer cells and fibroblasts.

## Methods

### Reagents

Recombinant human HGF was purchased from PeproTech (Rocky Hill, NJ). Anti-β-actin (Sigma-Aldrich, St. Louis, MO), anti-c-Met (IBL Co., Ltd., Gunma, Japan; #18321), and anti-phosphorylated c-Met (IBL Co., Ltd. #28083) antibodies were used. An anti-human HGF neutralizing antibody was also used (R&D Systems, Minneapolis, MN; AB-294-NA). A specific c-Met inhibitor, PHA-665752, was purchased from Sigma-Aldrich.

### Cell culture and collection of cell-conditioned medium

Human fetal lung fibroblasts (HFL1, MRC5 and IMR90 cells) were purchased from the American Type Culture Collection (Rockville, MD). Fibroblasts were cultured in DMEM supplemented with 10% FBS; cells between the 16th and 20th passages were used for experiments. Human lung cancer cell lines (A549 and HI1017, adenocarcinoma; EBC1, squamous cell carcinoma) were obtained from the Japan Cancer Research Bank (Tokyo, Japan). Lung cancer cells were cultured in RPMI-1640 supplemented with 10% FBS. To collect lung cancer cell- or fibroblast-conditioned medium, subconfluent cells were rinsed twice with serum-free DMEM and were then incubated with serum-free DMEM (at a density of 10^6^ cells/2 ml). After a 48-hour incubation, the medium was collected and centrifuged, and the supernatant was designated as “lung cancer cell-conditioned medium” or “fibroblast-conditioned medium” (e.g., “A549-medium” or “HFL1-medium”). Each type of cell-conditioned medium was prepared independently at least three times.

### Animals and xenotransplantation

Mice of the BALB/c-*nu* strain were purchased from Charles River Laboratories Japan, Inc. (Yokohama, Japan) and were maintained in the Division of Animal Experiments, Life Science Research Center, Kagawa University (Kagawa, Japan), according to the Institutional Regulations for Animal Experiments [15]. The protocols of the animal experiments were approved by the Animal Care and Use Committee at Kagawa University. For comparison of susceptibility to cancer cell engraftment, 10^5^ EBC1 cells with or without 10^5^ HFL1 or MRC5 cells were subcutaneously inoculated into 20 mice (10 mice each inoculated twice) when the mice were 6 weeks of age. The tumor sizes were measured every week with a caliper. The tumor volume (TV) was calculated using the formula TV = 1/2 × A × B^2^ (where A = length in millimeters and B = width in millimeters), as previously described [15, 16]. The criteria for successive engraftment were progressive nodule growth at the site of inoculation and tumor volumes greater than 10 mm^3^. Mice were monitored up to 8 weeks after inoculation at which time they were euthanized. For the experiments that required PHA-665752, after the onset of tumorigenesis, PHA-665752 (250 mM in 2% DMSO in PBS) or 2% DMSO (control) was injected around the EBC1-derived tumor once daily for a total of 10 days; this continued for 2 weeks. Mice were monitored for an additional week and then euthanized.

### Histology and immunohistochemistry

The engrafted tumors were fixed, stained with hematoxylin and eosin. The number of mitotic cells in microscopic 10 high power fields, ×400, (10 HPF) was counted. Immunohistochemical staining was performed according to the avidin-biotin complex (ABC) method. All staining processes from deparaffinization to counterstaining with hematoxylin were performed using the automated LEICA BOND-IIITM staining system (Leica Biosystems, Heidelberg, Germany). Antigen retrieval was not performed for α-SMA, but for vimentin, antigen retrieval was performed for 30 minutes by placing the sections in epitope retrieval buffer (pH 6) in the autostainer. The anti-α-SMA antibody (clone 1A4, code M0851, Dako, Glostrup, Denmark) was used at 1:150 dilution for a total reaction time of 15 minutes, while the anti-human multi-cytokeratin antibody (code NCL-L-AE1/AE3, Leica Biosystems) (1:300 dilution, 15 minutes) and the anti-human vimentin antibody (Clone V9; code M0725, Dako) (1:600 dilution, 15 minutes) were used to confirm the presence of human cell-derived tumors.

### Immunoblots

Immunoblots were performed as previously described [17]. Briefly, cells were lysed in lysis buffer (35 mM Tris [pH 7.4], 0.4 mM EGTA, 10 mM MgCl_2_, and 0.1% Triton-100) containing protease inhibitor and phosphatase inhibitor cocktails (Sigma-Aldrich). The total cell lysate was homogenized in 2× sodium dodecyl sulfate (SDS) sample buffer, boiled, subjected to SDS-polyacrylamide (10%) gel electrophoresis, and then transferred to a polyvinylidene difluoride membrane. The membrane was blocked with 1% BSA and incubated with the primary antibodies. After it was rinsed with 0.1% Tween-20 in PBS, the membrane was incubated with the appropriate HRP-conjugated secondary antibody. The intensity of the positive signals was visualized by chemiluminescence (GE Healthcare, Buckinghamshire, UK), and the images were imported by Image Reader LAS-1000 Plus (Fuji Photo Film Co. Ltd., Tokyo, Japan).

### Lung cancer cell survival

Lung cancer cell survival (viability) was assessed by WST-1 assay. Briefly, cells were incubated in regular medium with 10% FBS and 10% WST-1 reagent (Roche Applied Science, Mannheim, Germany). After incubation for 4 hours, 100 μl of each sample was transferred to a 96-well plate, and the absorbance at 450 nm was measured by a microplate reader (iMark^TM^, BIO-RAD). All samples were assessed at least in triplicate.

### Transwell co-culture of lung cancer cells with lung fibroblasts

Lung cancer cells were plated in a 6-well plate at a density of 2 × 10^5^ cells/well. The following day, the medium was replaced with serum-free DMEM. The lung fibroblasts were co-cultured separately on track-etched polyethylene terephthalate membrane inserts (1 μm pore size; Corning, Inc. NY, #353102), which allowed for interaction among the cells via soluble factors [18]. Cells inside the inserts were repeatedly cultured over a 7-day cycle; that is, the cells were cultured in 10% FBS for 2 days and in serum-free DMEM for 5 days. During culture in 10% FBS, the inserts were removed from the 6-well plate, after which the lung cancer cells were cultured under serum-free conditions. A WST-1 assay was used to determine cell survival (viability).

### HGF measurement by ELISA

The HGF concentration was measured using an ELISA kit (KAC2211, Life Technologies, Carlsbad, CA) according to the manufacturer’s instructions.

### Statistical analysis

Each experiment was repeated at least three times. Student’s t-test was used to compare data between two groups. The differences in tumorigenicity in each condition were assessed by Fisher’s direct probability method. *p* values < 0.05 were considered statistically significant. All statistical analyses were performed using Ekuseru-Toukei 2015 (Social Survey Research Information, Tokyo, Japan).

## Results

### Lung fibroblasts enhance the tumorigenicity of lung cancer cells

**Fig. 1:**
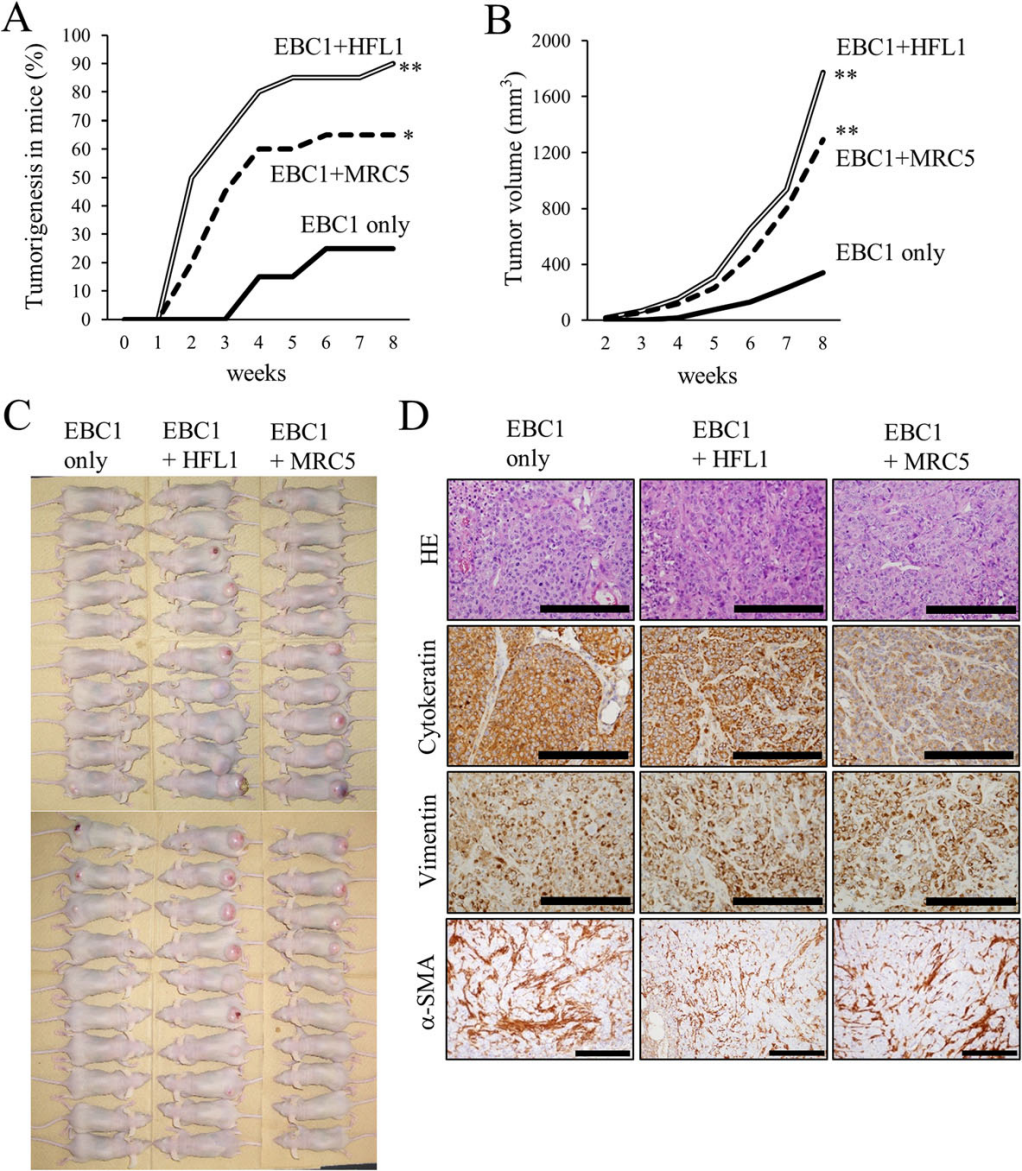
Lung fibroblasts enhance tumorigenicity in mice. **a** Tumorigenicity in BALB/c-*nu* mice after subcutaneous inoculation with 10^5^ EBC1 cells with or without 10^5^ FHL1 or MRC5 cells. *p < 0.05 or **p < 0.01, compared with EBC1 cells only. **b** Estimated tumor size. **p < 0.01, compared with EBC1 cells only. **c** At 8 weeks after inoculation, mice were euthanized and photographs were taken. **d** Histology of tumors that developed after inoculation of the mice with EBC1 with or without fibroblasts. HE: hematoxylin and eosin staining. SMA: smooth muscles actin. *Scale bars*: 200 μm. It is noteworthy that currently available anti-α-SMA antibodies are not specific for human cells

EBC1 cells were subcutaneously inoculated into BALB/c-*nu* mice with or without the same number of lung fibroblasts (HFL1 or MRC5 cells). When the mice were inoculated with EBC1 cells alone, tumorigenesis was observed in 5 of 20 mice (25%) at 8 weeks after inoculation (Fig. 1a). When EBC1 cells were co-implanted with fibroblasts, the tumorigenicity significantly increased (18 of 20 (90%) with HFL1 cells or 13 of 20 (65%) with MRC5 cells). As tumors developed and grew progressively, the tumor size at 8 weeks was larger after the co-implantation of tumor cells and fibroblasts (Fig. 1b and c). The pathologic findings of the developed tumors revealed squamous cell carcinoma, as expected (Fig. 1d). The tumor was positive for human cytokeratin and human vimentin, which suggests that the developed tumor originated from a human source, that is, EBC1 cells. Interestingly, no obvious difference was observed in vimentin and α-SMA positivity in the tumor stroma between tumors derived from EBC1 cells only and those derived from EBC1 cells with HFL1 or MRC5 cells at 8 weeks (data not shown) or at an even earlier time point (at 2 weeks) after inoculation (Fig. 1d).

### Lung fibroblast-conditioned medium contributes to lung cancer cell survival

**Fig. 2:**
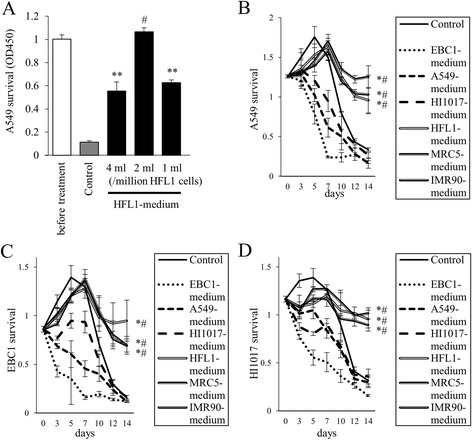
The survival of lung cancer cells is prolonged when cultured in lung fibroblast-conditioned media. **a** HFL1-media from three different conditions were collected (1, 2, or 4 ml of medium per one million HFL1 cells). 5 × 10^4^ A549 cells/well were seeded in 24-well plates. The following day, cancer cells were cultured in fresh serum-free medium (Control), HFL1-conditioned medium. After 2 weeks, cell survival (viability) was assessed by WST-1 assay. **p < 0.01, compared with fresh medium. ^#^ *p* < 0.01, compared with other conditions using HFL1-medium. **b**–**d** Time course of cancer cell **b**: A549, **c**: EBC1, and **d**: HI1017) survival in fibroblast- or lung cancer cell-conditioned media. Doubled lines indicate the three types of fibroblast-conditioned media. Dotted lines indicate lung cancer cell-conditioned media. *p < 0.05, compared with control (fresh serum-free medium). ^#^ *p* < 0.05, compared with the same type of cancer cell-conditioned medium

We next investigated the effect of lung fibroblast-conditioned medium on lung cancer cell survival. When A549 cells were cultured in serum-free medium, most cells died within 2 weeks (Fig. 2a). Interestingly, more than half of the cells survived when they were cultured in HFL1-medium. The most optimal HFL1-medium for the extension of cancer cell survival was prepared by the addition of 2 ml medium per one million HFL1 cells (Fig. 2a). This condition (2 ml of medium per one million cells) was the most effective for the promotion of the survival of A549 cells in MRC5-medium and IMR90-medium as well as in HFL1-medium (data not shown). Fig. 2b shows the time course of A549 cell survival when the cells were cultured in fibroblast-conditioned media derived from the 3 fibroblasts or lung cancer cell-conditioned media derived from the 3 lung cancer cell lines (A549, EBC1 and HI1017 cells). A549 cells died at an earlier time point when they were cultured in cancer cell-conditioned media than when they were cultured in fresh medium (control), whereas fibroblast-media from all 3 fibroblasts prolonged A549 cell survival. Similar to A549 cells, EBC1 cells and HI1017 cells were more likely to survive in fibroblast-conditioned media than in cancer cell-conditioned media or in control conditions (Fig. 2c and d, respectively).

### Co-culture with lung fibroblasts enhances lung cancer cell survival

**Fig. 3:**
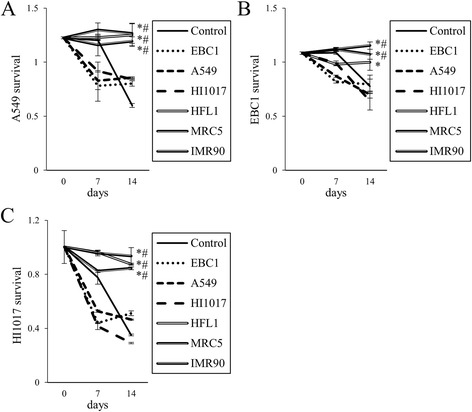
Prolonged lung cancer cell survival by co-culture with lung fibroblasts. Using the Transwell co-culture system, lung cancer cells (**a**: A549, **b**: EBC1 and **c**: HI1017) were cultured in the bottom wells in serum-free conditions. Lung cancer cells were co-cultured with either cancer cells or fibroblasts, which were seeded into the upper wells (on Transwell co-culture inserts). Inserts (without cells) were also prepared and served as controls. To maintain active conditions in cells in the upper wells, the inserts were removed, and the cells were cultured in 10% FBS for 2 days and rinsed with serum-free medium. The inserts were then placed again in the co-culture system, and the cells were cultured in serum-free medium for 5 days; this 7-day culture cycle was then repeated. Cell survival (viability) was assessed by WST-1 assay. Doubled and dotted lines indicate co-culture with fibroblasts and cancer cells, respectively. *p < 0.05, compared with control (without co-culture). ^#^ *p* < 0.05, compared with co-culture with the same type of cancer cells

To confirm the enhancement of lung cancer cell survival by lung fibroblasts, A549, EBC1 and HI1017 cells were co-cultured with fibroblasts separately in a Transwell co-culture system (Fig. 3). When co-cultured with fibroblasts, the cancer cells were more likely to survive in serum-free medium compared with cells without co-culture and cells co-cultured with cancer cell lines.

### HGF produced by lung fibroblasts contributes to cancer cell survival

**Fig. 4:**
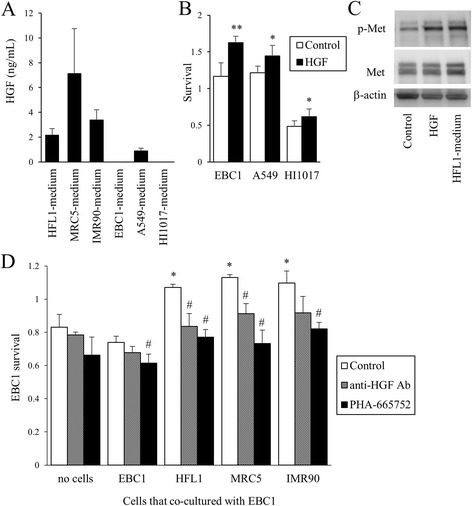
The effect of HGF produced by lung fibroblasts on cancer cell survival. **a** Production of HGF by lung cancer cells and fibroblasts in the culture media. Cells were cultured in serum-free conditions (2 ml/one million cells) for 2 days, and media were collected. The HGF concentration was measured by ELISA. **b** Lung cancer cell survival with or without exogenous HGF (10 ng/ml). After a 7-day incubation in culture medium with 1% FBS, cell survival (viability) was assessed by WST-1 assay. *p < 0.05, **p < 0.01, compared with the control. **c** Immunoblots. EBC1 cells were stimulated with HGF or cultured in HFL1-medium for 1 hour, and total cell lysates (10 μg/lane) were immunoblotted for Met and phosphorylated Met (p-Met). **d** EBC1 cell survival. Cells were cultured in fibroblast-conditioned media with anti-HGF neutralizing antibody or PHA-665752 (100 nM) for 14 days, and WST-1 was performed. *p < 0.05, compared with no co-culture. ^#^ *p* < 0.05, compared with control (no anti-HGF antibody or PHA-665752)

HGF was produced to a greater extent by the three fibroblasts (2.2, 7.1, and 3.4 ng/mL in HFL1-, MRC5-, and IMR90-media, respectively) than by lung cancer cells (Fig. 4a). Exogenous HGF increased the survival of lung cancer cells (Fig. 4b) and stimulated the phosphorylation of Met, the receptor of HGF, as predicted (Fig. 4c). HFL1-medium also stimulated the phosphorylation of Met expression. The prolonged survival of cancer cells induced by co-culture with fibroblasts was abolished in the presence of either anti-HGF neutralizing antibody or PHA-665752, a specific Met inhibitor (Fig. 4d).

### Inhibition of Met suppresses the progression of EBC1-derived tumors in a xenograft model

**Fig. 5:**
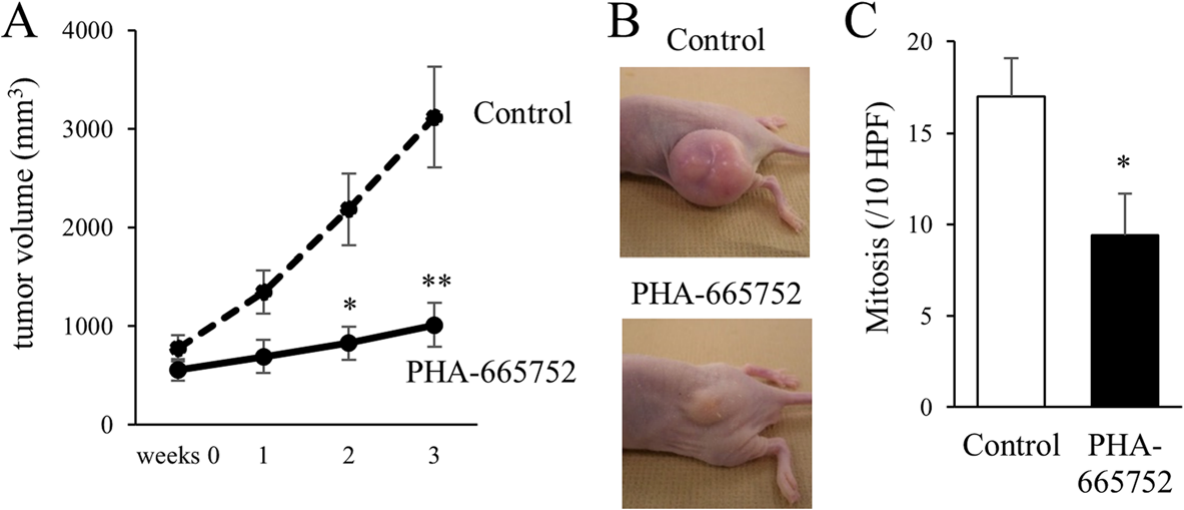
The effect of PHA-665752 on tumor progression. **a** EBC1 and HFL1 cells were co-inoculated into BALB/c-*nu* mice. After the confirmation of tumorigenesis, 100 μl of PHA-665752 (250 μM) or vehicle control (2% DMSO in PBS) was injected in the area surrounding the tumor once a day for 10 days over a period of 2 weeks; the tumor volumes were estimated every week. Seven tumors were assessed in each group. Data are expressed as the means ± SEM of seven tumors. *p < 0.05, **p < 0.01, compared with the control. **b** Representative tumor derived from EBC1 cells in mice at 3 weeks from the initiation of treatment. **c** Mitotic rates of cancer cells in the tumors. Three tumors from each condition were resected and stained, and the number of mitotic figures was counted. *p < 0.05 compared with the control

We next investigated the effect of the inhibition of Met on tumor progression in a mouse xenograft model. After tumor formation, which occurred after the co-inoculation of mice with EBC1 cells and HFL1 fibroblasts, PHA-665752 was injected into the areas surrounding the tumor daily for a total of 10 days over a period of 2 weeks. The application of PHA-665752 resulted in significant inhibition of tumor progression (Fig. 5a and b). The number of mitotic figures in the tumors treated with PHA-665752 was significantly lower than that in the control (Fig. 5c).

### Lung cancer cell-conditioned medium stimulates the production of HGF from lung fibroblasts

**Fig. 6:**
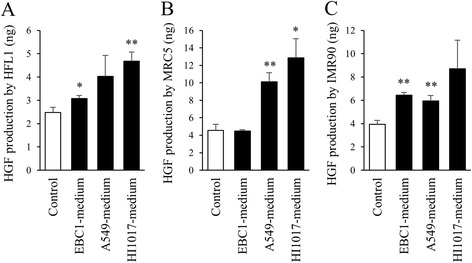
HGF production in fibroblasts cultured in lung cancer cell-conditioned media. Lung fibroblasts (**a**: HFL1, **b**: MRC5, **c**: IMR90) were incubated in fresh medium (control) or lung cancer cell-conditioned media. After 2 days, conditioned media were collected, and the HGF concentration was measured by ELISA. Data are expressed as the means of concentration (ng/ml) per million cells ± SEM of three separate experiments. *p < 0.05, **p < 0.01, compared with the control

To assess whether the production of HGF by lung fibroblasts is affected by lung cancer cells, lung fibroblasts were cultured in lung cancer cell-conditioned media for 2 days, and then the culture media were collected. Interestingly, when cultured in lung cancer cell-conditioned media, lung fibroblasts produced HGF to a greater extent than when they were cultured in control conditions (i.e., regular fibroblast-conditioned media) (Fig. 6). It was noted again that the HGF concentration in lung cancer cell-conditioned media was lower than that in regular fibroblast-conditioned media (Fig. 4).

## Discussion

**Fig. 7:**
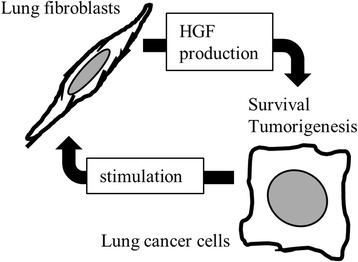
Schema of the interaction between lung cancer cells and lung fibroblasts. Lung fibroblasts enhance lung cancer cell survival and tumorigenesis via the production of HGF. Lung cancer cells stimulate HGF production in lung fibroblasts.

In the present study, we demonstrated the following: (1) co-inoculation of mice with lung cancer cells and lung fibroblasts results in higher susceptibility to tumorigenesis and higher tumor progression in those mice, (2) lung fibroblast-conditioned medium contributes to lung cancer cell survival through, at least in part, the production of HGF, (3) inhibition of HGF/Met signaling can suppress cancer cell survival and tumor progression, and (4) lung cancer cells stimulate HGF production in lung fibroblasts, as summarized in Fig. 7.

In the present study, all three tested lung fibroblast-conditioned media led to a prolonged survival of lung cancer cells. Consistent with this, in a 3D co-culture model, the co-culture of MRC5 fibroblasts and cancer cell lines increased the survival of several types of cancer cells [1]. In lung cancer, however, only 2 of the 7 cell lines tested exhibited increased survival when co-cultured with MRC5 cells for 5 days, and no increased survival was observed in A549 cells [1]. In the present study, however, MRC5-conditioned medium clearly prolonged the survival of A549 cells. One possible reason for this difference is that the culture period was different, which may be important because in the present study, the effect of fibroblast-conditioned medium was more obvious after one week (Fig. 2).

The co-inoculation of mice with lung cancer cells and lung fibroblasts increased the incidence of tumorigenesis and promoted tumor progression. The fibroblasts surrounded by cancer cells tested in the current study are akin to CAFs. A two-week period seems to be sufficient for the effect of fibroblasts on the stimulation of cancer progression to be observed. Interestingly, it has been reported that the subcutaneous co-inoculation of SCID mice with A549 cells and CAFs significantly enhanced tumor growth compared with co-inoculation with normal fibroblasts [19]. Although we attempted to detect co-inoculated fibroblasts within the tumor, no significant difference was found in the stroma between the conditions with or without co-inoculation with fibroblasts even at 2 weeks after co-inoculation. This might be due to the difference in cell proliferation rates between cancer cells and fibroblasts; that is, cancer cells grow very rapidly so that fibroblasts are soon outnumbered. Another possibility is that lung fibroblasts might be induced by lung cancer cells to undergo apoptosis.

The present study suggests that the strategy of lung cancer treatment should include the regulation of either fibroblasts or some of the factors produced by fibroblasts, such as HGF. In this regard, the effects of the inhibition of HGF/Met signaling on lung cancer progression have been reported. PHA-665752 has been shown to reduce the tumorigenicity of lung cancer cell lines (NCI-H69 and NCI-H441) in mouse xenografts [10]. In the current study, PHA-665752 also significantly suppressed tumor progression after the co-injection of EBC1 and HFL1 cells. One mechanism for the inhibition of tumor progression might be the occurrence of cell cycle arrest, as evidenced by a decrease in mitosis. PHA-665752 also reportedly inhibits the formation of vascular structures [10].

In the treatment of NSCLC cases that harbor *epidermal growth factor receptor* (*EGFR*) mutations, EGFR-tyrosine kinase inhibitors (TKIs), such as gefitinib, erlotinib, and afatinib, can block the survival signals that are mediated by this driver oncogene and can induce marked tumor regression [14]. However, tumors eventually acquire resistance to EGFR-TKIs and regrow in almost all cases. The mechanisms of EGFR-TKI resistance include alterations of the *EGFR* gene (e.g., T790M mutation) and activation of bypass signaling pathways (e.g., *Met* gene amplification and HGF overexpression). Indeed, in one study, exogenous HGF induced resistance to an anti-EGFR antibody in lung cancer cells (via the Met/Gab1/Akt signaling pathway) [11]. In addition, resistance to an anti-EGFR antibody was also induced in tumor cells by the co-culture of cancer cells and HGF-producing lung fibroblasts [11]. Moreover, the usefulness of the inhibition of the HGF/Met pathway for the treatment of EGFR-TKI-resistant lung cancer cells has been reported [9, 12, 13]. The Met inhibitor SU11274 exerted a pro-apoptotic effect on EGFR-TKI-resistant H1975 cells and induced tumor regression in H1975 xenografts [9]. The combination of SU11274 and erlotinib treatment induced complete tumor regression in a xenograft model [9]. E7050, a dual inhibitor of Met and vascular endothelial growth factor receptor two kinase, and EGFR-TKIs suppressed tumor progression in erlotinib-resistant cancer cell lines and in HGF-induced EGFR-TKI-resistant lung cancer cell lines that also have a mutation in *EGFR* [12, 13].

Two phase III clinical trials using a Met inhibitor tivantinib (ARQ 197) and erlotinib have been conducted [20, 21]. The combination of erlotinib plus tivantinib increased the progression-free survival of patients with previously treated advanced non-squamous NSCLC [21]. In a subgroup of patients with high Met expression, tivantinib also improved the overall survival. However, another trial targeting Asian patients with EGFR-WT non-squamous NSCLC was prematurely terminated due to the increased incidence of interstitial lung disease in patient group treated with erlotinib and tivantinib, although preliminary data revealed that the PFS was longer in that group [20]. *Met* amplification is an excellent predictor of sensitivity to Met inhibitors such as PHA-665752 and crizotinib [22, 23]. Impaired Met degradation mediated by *Met* exon 14 mutations has also been documented in NSCLC [24–26]. *Met* exon 14 skipping was identified in 2.7% of patients with NSCLC, and a robust response to crizotinib was observed regardless of the *Met* amplification status [26]. Ethnicity and Met activation as a predictive marker (e.g., Met expression, *Met* amplification or *Met* exon 14 skipping) should be considered prior to the clinical application of Met inhibitors.

Another important finding of the current study is that HGF production in fibroblasts was stimulated by lung cancer cells, which seemed to exploit the fibroblasts for their own growth. Unfortunately, we could not identify the factor that stimulates HGF production in fibroblasts. Although transforming growth factor-β is known to change the characteristics of lung fibroblasts and is produced by lung cancer cells, its exogenous addition did not stimulate HGF production by fibroblasts (data not shown). The identification of the specific factor that is released from lung cancer cells that stimulates HGF production by fibroblasts might also be useful for the development of a novel strategy for lung cancer treatment.

## Conclusions

The current study showed that lung fibroblasts promote lung cancer cell survival, tumorigenicity and tumor progression via HGF/Met signaling. This signaling pathway might therefore serve as a therapeutic target that involves an anti-CAF strategy.

## Abbreviations

CAF: Cancer-associated fibroblast
EGFR: Epidermal growth factor receptor
HGF: Hepatocyte growth factor
NSCLC: Non-small cell lung cancer
TKI: Tyrosine kinase inhibitor
